# Relapsing anemia associated with parvovirus B19 infection in a kidney transplant recipient: A case report and review of the literature

**DOI:** 10.1002/ccr3.7906

**Published:** 2023-09-07

**Authors:** Fatemeh Yaghoubi, Davood Dalil, Farnaz Tavakoli, Seyyed Mohammad Hosseini

**Affiliations:** ^1^ Nephrology Research Center, Shariati Hospital Tehran University of Medical Sciences Tehran Iran; ^2^ Student Research Committee, Faculty of Medicine Shahed University Tehran Iran

**Keywords:** anemia, ESRD, kidney transplant, parvovirus B19

## Abstract

**Key clinical message:**

PB19 infection should be considered an uncommon cause of posttransplant anemia in renal transplant recipients, particularly those whose anemia is not associated with common etiologies. IVIG treatment and reduced immunosuppression could be beneficial.

**Abstract:**

Parvovirus B19‐associated relapsing anemia is rare in kidney transplant recipients. Herein, we report a case of relapsed anemia due to parvovirus B19 infection in a 53‐year‐old woman 18 months after kidney transplantation. The patient presented with palpitations, shortness of breath, dizziness, weakness, and lethargy. Early laboratory findings showed a WBC count of 6.000/μL, RBC count of 1.89/μL, hemoglobin (Hb) 3.5 g/dL, hematocrit (Hct) 15%, platelet count 266.000/μL, MCV 89, reticulocyte count 0.8%, and serum iron 221 μg/dL. Upon further evaluation, the RT‐PCR test for BK polyomavirus and cytomegalovirus (CMV) was negative, while the parvovirus B19 RT‐PCR was positive. The patient was treated with blood transfusion and IVIG 25 g daily for 5 days. Two months after discharge, the patient presented, complaining of palpitation, shortness of breath, and dizziness, with RBC 2.7/μL, Hb 6.5 g/dL, Hct 25%, and MCV 85. Again, the CMV RT‐PCR was negative, while the parvovirus B19 RT‐PCR was positive. Tacrolimus and mycophenolic acid were stopped, and IVIG 25 g daily for 5 days was administered. Consequently, her Hb level increased to 9 g/dL, and the patient was discharged with prednisolone 5 mg daily and cyclosporine 50 mg daily instead of tacrolimus. Viral infection, particularly PB19 infection, should be considered in the differential diagnosis of posttransplantation anemia in KTRs. IVIG treatment and modification of immunosuppressive medications are suggested standard therapies for such patients. The function of transplanted kidneys should be carefully monitored during treatment.

## 1 INTRODUCTION

Anemia is a posttransplant complication reported in 25%–50% of kidney transplant recipients (KTRs). It is a significant risk factor for the leading cause of death, cardiovascular disease, among KTRs.^1^ Kidney transplant patients are more prone to infections due to taking immunosuppressive (IS) drugs. Parvovirus B19 (PB19), a single‐stranded DNA virus of the *Parvoviridae* family,^2^ has been reported to infect up to 65% of KTRs following transplantation. Anemia is a rare but expected finding of PB19 infection in KTRs due to the lysis of erythroid progenitor cells (EPCs) through viral lytic replication.^3^ Intravenous immunoglobulin (IVIG) treatment (administration of PB19‐IgG antibodies) is shown to be a successful treatment for PB19‐associated anemia. However, relapsing anemia related to PB19 is highly uncommon, occurring in almost 33% of patients treated with IVIG.^4^ Here, we presented in detail a case of PB19‐associated relapsing anemia in a KTR woman 18 months after transplantation. Further, we reviewed the pertaining literature during the past decade.

## 2 CASE PRESENTATION

The patient was a 53‐year‐old woman who presented to Shariati Hospital, Tehran, Iran, with complaints of palpitations, shortness of breath, dizziness, weakness, and lethargy. The patient did not complain of nausea, vomiting, cough, fever, or oliguria. She was a known case of end‐stage renal disease (ESRD) of unknown origin and underwent a kidney transplant from a deceased donor 18 months prior. Her medical history included hypothyroidism, hypertension, and two episodes of preeclampsia, which resulted in preterm labor. The patient was on prednisolone 7.5 g daily, tacrolimus 1.5 g BD, mycophenolic acid 360 mg BD, levothyroxine 0.1 mg daily, carvedilol 6.25 mg BD, furosemide 20 mg BD, sitagliptin/metformin 50/500 mg BD, and Nephrovite daily.

Early laboratory findings showed a white blood cell (WBCs) count of 6.000/μL, red blood cells (RBCs) count of 1.89/μL, hemoglobin (Hb) 3.5 g/dL, hematocrit (Hct) 15%, platelet count 266.000/μL, mean corpuscular volume (MCV) 89, reticulocyte count 0.8%, erythrocyte sedimentation rate (ESR) 119 mm/h, C‐reactive protein (CRP) 49 mg/L, lactate dehydrogenase (LDH) 724 IU/L, creatine phosphokinase (CPK) 26 IU/L, blood urea nitrogen (BUN) 38 mg/dL, creatinine (Cr) 2.5 mg/dL, albumin 3.7, Ca 8.5, P 3.4, bilirubin (total: 1.1 and direct: 0.32 mg/dL), serum iron (SI) 221 μg/dL, total iron‐binding capacity (TIBC) 200 μg/dL, ferritin more than 1200 ng/mL. Serum and urine protein electrophoresis (SPEP/UPEP) results were normal, vitamin B12 and folate serum levels were normal, and the direct and indirect Coombs test was negative. Real‐time polymerase chain reaction (RT‐PCR) for BK polyomavirus and cytomegalovirus (CMV) was negative, while the PB19 RT‐PCR was positive.

The patient was treated with a blood transfusion and IVIG (25 g daily for 5 days). Thus, she was discharged with Hb 8.6 g/dL and home‐use medicines of erythropoietin, ferrous sulfate, and Nephrovite. Three weeks later, during her clinical follow‐up, she was clinically stable with Hb 10.8 g/dL, Cr 1.5 mg/dL, and a negative RT‐PCR test of PB19. Two months after hospitalization, the patient was referred to the emergency department of our hospital with complaints of palpitations, shortness of breath, and dizziness. Her vital signs were normal (pulse rate, 90 beats/min; respiratory rate, 12 breaths/min; blood pressure, 130/80 mmHg). The blood tests revealed WBC 8.000/μL, RBC 2.7/μL, Hb 6.5 g/dL, Hct 25%, MCV 85, platelet count 300.000/μL, Cr 2 mg/dL, and other biomarkers were normal. Again, the CMV RT‐PCR result was negative, whereas the PB19 RT‐PCR result was positive.

In the clinical management of the patient, the drugs that she normally used were first modified. Treatment with tacrolimus, mycophenolic acid, sitagliptin, and metformin was discontinued. IVIG (25 g daily for 5 days) was administered. Consequently, her Hb and Cr levels were 9 and 2 mg/dL, respectively. The patient was discharged with prednisolone 5 mg daily, cyclosporine 50 mg in the morning and 75 mg at night daily, epoetin alfa 5000 UI three times a week, linagliptin 5 g daily, and ferrous sulfate/folic acid 60/0.4 mg daily.

## 3 DISCUSSION

KTRs are more sensitive to infectious diseases, especially during the first year after transplantation, due to the use of IS drugs, such as anti‐thymocyte globulin (ATG), to prevent acute rejection and maintain an IS regimen to prevent graft failure. PB19 has been demonstrated as one of the most common opportunistic viral infections during the first year after renal transplantation, right behind CMV and EBV.^5^ PB19 tendentiously infects human EPCs, replicates them, and then lyses the EPCs.^6^, ^7^ Erythropoiesis pauses for approximately 1 week during the viremic phase, with no significant clinical manifestations. It begins again with the production of neutralizing antibodies against the virus.^8^

However, immunocompromised patients may experience persistent infection owing to insufficient antiviral antibody production, leading to pure red blood cells aplasia (PRCA).^9^ Fever, rash, arthralgia, normochromic normocytic anemia, and lack of response to erythropoietin are the most common manifestations of PB19 infection in immunosuppressed patients.^10^ PB19 infection may involve other organs, leading to myocarditis, pneumonitis, hepatitis, and graft failure.^3^, ^11^ In our patient, the clinical suspicion of erythrocyte‐involving infection, such as PB19, was raised due to the decrease in erythrocyte levels as well as the lack of evidence of common causes of anemia, including gastrointestinal or urinary bleeding, hemolysis, or malnutrition.

Respiratory tract infections, blood transfusions, viral reactivation, and transplanted organs are possible routes of PB19 transmission in KTRs. Capenko et al. investigated the prevalence of PB19 infection among renal transplant donors and recipients and revealed that an active viral infection remarkably caused severe anemia posttransplantation anemia.^12^ The risk factors associated with PB19 infection must be taken into account to help the early detection of the cause of anemia in KTRs. Boaek et al. detected the correlation between kidney graft from a deceased donor, tacrolimus treatment, and low Hb levels with the PB19 infection within the first year post‐renal transplantation.^13^ Laboratory methods that directly detect the virus, such as RT‐PCR, are more sensitive than antibody assays recommended for immunocompromised patients.^14^ If RT‐PCR is negative, but there is still strong clinical suspicion, bone marrow biopsy can aid in the diagnosis by showing giant pronormoblasts with inclusion bodies.^3^ In our case, the suspicion of PB19 infection was confirmed by RT‐PCR at both times.

Passive immunization with IVIG treatment has been shown to be effective in first‐line management of PB19‐related PRCA. It is usually recommended to start IVIG treatment at an initial dose of 400 mg/kg for at least 5 days, which can be repeated if the infection recurs.^15^, ^16^ The second line in the management of KTR patients with PB19‐related anemia is the modification of their used IS regimen. Nevertheless, there is a risk of acute rejection of kidney graft in the case of reduced immunosuppression particularly during the first year after transplantation. Discontinuation of tacrolimus or switching to cyclosporine was suggested as part of the treatment strategy. Moudgil et al. presented a case of PB19‐related graft failure in a KTR patient whose infection was cured after the cessation of tacrolimus.^17^ Wong et al. reported the failure of repeated courses of IVIG therapy for PRCA associated with PB19 infection in a KTR treated with tacrolimus.^18^ Furthermore, several studies have shown rapid improvement after substituting tacrolimus with cyclosporine in patients with tacrolimus‐mediated PRCA.^15^, ^19^, ^20^ The patient's Hb level would improve on average after 4 weeks. Our patient was treated with 25 g IVIG for 5 days, cessation of tacrolimus and mycophenolic acid, and using cyclosporine. His Hb level was almost normal after 3 weeks.

During the past decade, several studies have reported persistent or recurrent anemia related to PB19 infection in KTRs which are different in some aspects of clinical course, treatment, and IS medications. Altheaby et al. presented the three cases of relapsing sever normochromic normocytic anemia (Hb <6 mg/dL) due to PB19 infection diagnosed by positive RT‐PCR during the first 6 months after renal transplantation. They found IVIG treatment in conjunction with limiting IS medication to be beneficial.^21^ Persistent unexplained anemia for 2 months after living kidney transplantation was diagnosed to be related to PB19 infection, confirmed by RT‐PCR and bone marrow biopsy. Three weeks IVIG treatment resolved the anemia completely.^10^ Tiong et al. reported a severe complication, splenic rupture, following IVIG treatment for persistent anemia due to PB19 infection. A splenectomy was performed and PB19‐infected cells were identified within the spleen.^22^

Inoue et al. demonstrated a donor‐transmitted PB19 infection that caused a refractory anemia posttransplantation. The PB19 infection of allograft was confirmed by immunoperoxidase staining of renal tubules and remission resulted after administration of standard therapy.^23^ Kaya et al. presented a case of severe recurrent PRCA (with Hb 5 mg/dL) three times over 1 year after transplantation without renal dysfunction who responded well to IVIG and change of tacrolimus to cyclosporine.^24^

Gang et al. reported a case of recurrent PB19‐related PRCA during 1‐year post‐renal transplantation, with 7 months duration between two anemic episodes. Although reducing tacrolimus with IVIG treatment improved the clinical condition at the first episode of anemia, ultimately switching from tacrolimus to cyclosporine improved the anemia and decreased the PB19 viral load.^25^ Changing from tacrolimus to everolimus, a mTOR inhibitor, was found favorable for a KTR with recurrent anemia associated with chronic PB19 infection who did not first benefit from standard therapy.^26^ However, Nowacka‐Cieciura et al. showed that the effectiveness of everolimus requires more studies. They reported recurrent PB19‐related PRCA in a woman with simultaneous pancreas and renal transplantation. After improvement of the first anemic episode with discontinuation of mycophenolate mofetil, red‐cell transfusions, and subcutaneous immunoglobulins, they added low doses of everolimus to benefit its antiviral potential. But, 3 months later the PRCA relapsed and after withdrawal of everolimus and administration of the standard therapy the patient improved completely.^27^

Our case report may highlight significant implications in the clinical management of KTR patients with PB19‐related relapsing anemia. The screening for PB19 in kidney transplant donors and recipients can prevent posttransplant infections and consequent complications. The baseline immunosuppression protocol of KTR should be lowered in such patients to benefit the potential of the immune system against PB19 infection. The cellular immune response is essential to establish protective immunity against PB19. However, the IS regimen should be modified based on the patient's medical history, clinical observation, and case‐by‐case to control the risk of graft rejection. In cases of recurrent PB19 infection, the patients should be monitored closely after the first treatment with IVIG to provide protection from recurrence as the IVIG effects vanish. The decision about the role of IS drugs such as tacrolimus in developing the PB19‐related PRCA requires more experimental and clinical studies. In conclusion, Physicians should be aware of PB19 infection as an underestimated cause of PRCA in KTRs post‐renal transplantation irrespective of IS protocol or types of donor.

## AUTHOR CONTRIBUTIONS

**Fatemeh Yaghoubi:** Conceptualization; investigation; project administration; writing – review and editing. **Davood Dalil:** Data curation; software; supervision; writing – original draft; writing – review and editing. **Farnaz Tavakoli:** Conceptualization; investigation; writing – review and editing. **Seyyed Mohammad Hosseini:** Writing – original draft; writing – review and editing.

## FUNDING INFORMATION

The authors received no financial support for this study.

## CONFLICT OF INTEREST STATEMENT

The authors declare that they have no conflicts of interest.

## ETHICS STATEMENT

The Research Ethics Committee of Tehran University of Medical Sciences approved all procedures performed in the current study with the approval number: IR.TUMS.SHARIATI.REC.1402.013. The patient expressed her informed consent through a written form.

## CONSENT

Written informed consent was obtained from the patient to publish this report in accordance with the journal's patient consent policy.

## Data Availability

None.
